# Clinical significance of part-solid lung cancer in the eighth edition TNM staging system

**DOI:** 10.1093/icvts/ivab255

**Published:** 2021-09-25

**Authors:** Tatsuro Okamoto, Michiyo Miyawaki, Gouji Toyokawa, Takashi Karashima, Miyuki Abe, Yohei Takumi, Takafumi Hashimoto, Atsuhi Osoegawa, Tetsuzo Tagawa, Hideya Takeuchi, Mototsugu Shimokawa, Kenji Sugio

**Affiliations:** Department of Thoracic and Breast Surgery, Oita University Faculty of Medicine, Oita, Japan; Department of Surgery and Science, Kyushu University Graduate School of Medicine, Fukuoka, Japan; Department of Thoracic and Breast Surgery, Oita University Faculty of Medicine, Oita, Japan; Department of Surgery and Science, Kyushu University Graduate School of Medicine, Fukuoka, Japan; Department of Thoracic and Breast Surgery, Oita University Faculty of Medicine, Oita, Japan; Department of Thoracic and Breast Surgery, Oita University Faculty of Medicine, Oita, Japan; Department of Thoracic and Breast Surgery, Oita University Faculty of Medicine, Oita, Japan; Department of Thoracic and Breast Surgery, Oita University Faculty of Medicine, Oita, Japan; Department of Thoracic and Breast Surgery, Oita University Faculty of Medicine, Oita, Japan; Department of Surgery and Science, Kyushu University Graduate School of Medicine, Fukuoka, Japan; Department of Surgery and Science, Kyushu University Graduate School of Medicine, Fukuoka, Japan; Department of Thoracic and Breast Surgery, Oita University Faculty of Medicine, Oita, Japan; Biostatistics Laboratory, Clinical Research Institute, National Hospital Organization Kyushu Cancer Center, Fukuoka, Japan; Department of Biostatistics, Yamaguchi University Graduate School of Medicine, Yamaguchi, Japan; Department of Thoracic and Breast Surgery, Oita University Faculty of Medicine, Oita, Japan

**Keywords:** Non-small-cell lung cancer, Staging system, Surgery, Prognosis, Part-solid tumour

## Abstract

**OBJECTIVES:**

The ground-glass component of part-solid tumour (PST) was eliminated as a clinical T (cT) descriptor in the eighth edition of the tumour, node and metastasis (TNM) staging system. We aimed to validate the new cT descriptor and investigate the prognostic impact of PST in the new staging system.

**METHODS:**

Non-small-cell lung cancer (NSCLC) patients (*n* = 1061) who underwent lung resection and were available for the assessment of thin-section computed tomography images were retrospectively reviewed. Tumours with a solid component (SC) size-to-whole tumour size (STR) ratio of 0, those with 0 < STR < 1 and those with an STR of 1 were defined as pure ground-glass tumours, PSTs and solid tumours (STs), respectively.

**RESULTS:**

Tumours with an SC diameter of >30 mm were less frequently observed among PSTs than among STs (4.83% vs 32.6%, *P* < 0.001). The postoperative 5-year survival of NSCLC patients with ground-glass tumour, PST and ST was 97.6%, 89.0% and 76.3%, respectively. In the survival analysis of patients with an SC diameter ≤30 mm, significant differences were observed among PST and ST (5-year survival, 90.7% vs 74.6%, *P* < 0.001). The multivariable analysis showed that age <70 years old, female sex, procedures with a lobectomy or more, SC size, pN0 disease and PST were independent predictors of a better survival among all PST and ST patients.

**CONCLUSIONS:**

Among patients with cT1 tumours, those with PST showed a significantly better survival than did those with ST. Small-sized PST tumours may not be suitable for the new cT descriptor.

**Figure:**
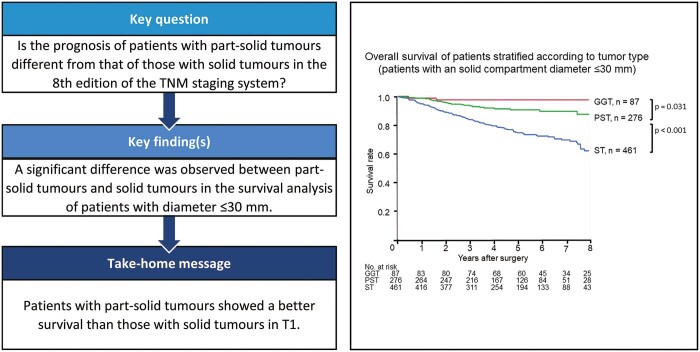

## INTRODUCTION

The tumour, node and metastasis (TNM) staging system has been one of the most practical and important prognostic predictors of lung cancer for several decades [1, 2]. Clinicians generally decide the therapeutic strategies for their patients with non-small-cell lung cancer (NSCLC) based on the clinical TNM stage. The recent revision of the eighth edition of the TNM staging system was made based on a large clinical database of 94 708 cases that were collected from 16 countries over the world [3]. Among the 3 descriptors of the staging system, the T descriptor was most greatly modified in the latest revision [4]. T1 and T2 were finely divided by 10 mm each for up to 50 mm in tumour diameter, and T3 and T4 were also re-categorized by tumour diameter as well as the extent of invasion. Moreover, the T descriptor assessment of part-solid tumours (PSTs) was dramatically changed in this revision, shifting from whole tumour diameter to solid component (SC) diameter [5]. In this revision, the ground-glass component (GGC) on thin-section computed tomography (CT) scan and the lepidic growth component of cancer cells in pathological specimens have been eliminated from the tumour diameter, based on findings that invasive size alone more precisely predicts patient survival [6, 7]. Although the new T descriptor has been practically used worldwide since January 2017, there are still limited data for validating the descriptor. Recently, several studies indicated that PST composed of both SC and GGC had a more indolent character than pure solid tumour (ST) regardless of the GGC size in the tumour [8, 9]. In the present study, we reviewed thin-section images of preoperative CT scans of patients with NSCLC in 2 institutions in Japan to investigate the prognostic significance of PSTs in the clinical T (cT) descriptor of the eighth edition of the TNM staging system.

## PATIENTS AND METHODS

### Patients

Consecutive NSCLC patients who underwent lung resection at Oita University Hospital between 2006 and 2014 (*n* = 828) and Kyushu University hospital between 2003 and 2012 (*n* = 710) were retrospectively reviewed. Patients who received any preoperative treatment were excluded. The complete clinical data of 1061 NSCLC patients were available for the assessment of thin-section CT images and prognostic analyses. The tumours included 809 adenocarcinomas, 197 squamous cell carcinomas, 31 large cell carcinomas, 18 adenosquamous carcinomas and 6 other types. The surgical procedures consisted of 298 sublobar resections, 746 lobectomies and 17 pneumonectomies. Nine hundred thirty-one patients had tumours that were pathologically proven to have no lymph node metastasis (pN0). Among N0 patients, the tumours included 730 adenocarcinomas, 163 squamous cell carcinomas, 20 large cell carcinomas, 12 adenosquamous carcinomas and 6 other types. The surgical procedures for N0 patients consisted of 292 sublobar resections, 627 lobectomies and 12 pneumonectomies. Histological diagnosis of tumour cell type was based on the World Health Organization histological classification of lung tumours (fourth edition, 2015). Basically, pulmonary lobectomy with hilar and mediastinal lymph node dissection was selected for lung cancer resection except for Tis or T1mi tumours. Sublobar resection was selected for Tis or T1mi tumours or for patients with limited pulmonary function for lobectomy according to a surgeon’s preference.

### Ethical statement

The institutional review boards of Oita University and Kyushu University approved the protocol of this study (approval no. 1567 and no. 24-173, respectively) with a waiver of informed patient consent.

### Evaluation of the computed tomography images

Preoperative thin-section CT scan findings were reviewed by M.M. and T.O. for patients of Oita University Hospital and by G.T. and T.O. for Kyushu University Hospital. If the independent assessments did not agree, it was reviewed together to achieve consensus. Tumour size was determined preoperatively based on these thin-section CT findings. The condition of CT examination and the measuring method were previously described [10]. Briefly, chest CT was performed in the supine position during inspiratory breath-hold using various multidetector row scanners: Aquilion 4, Aquilion 64, Aquilion ONE and Aquilion ONE VISION (Toshiba Medical Systems, Tochigi, Japan); Somatom Plus 4 Volume Zoom (Siemens Medical Solutions, Malvern, PA, USA); and Brilliance CT and Brilliance iCT (Philips, Amsterdam, Netherlands). The imaging parameters for thin-section CT were as follows: peak tube voltage 120 kVp; tube current 100–500 mA; scan field of view 320–360 mm; and slice thickness 1 mm (Oita University Hospital) or 2 mm (Kyushu University Hospital). All of the CT data sets were transferred to a Picture Archiving and Communication System (PACS). The diameter of the pulmonary tumour was measured manually using axial 2-dimensional CT data at a 1-mm slice section (Oita University Hospital) or 2-mm slice section (Kyushu University Hospital). The tumours with a ratio of SC size-to-whole tumour size (STR) = 0, those with 0 < STR < 1 and those with STR = 1 were defined as pure ground-glass tumours (GGTs), PSTs and STs, respectively (Supplementary Material, Fig. S1).

### Patient follow-up

Patients underwent physical examination; laboratory examination for blood cell counts, serum chemistry and serum tumour markers including carcinoembryonic antigen; and chest radiography every 3–6 months after the operation. The chest and upper abdominal CT scans were performed once or twice per year. Brain magnetic resonance imaging and bone scintigrams or fluorodeoxyglucose positron-emission tomography scans were performed annually. The median follow-up period was 5.1 years. A total of 196 patients dropped from the follow-up within 3 years after surgery. Two hundred twenty-two events were calculated in the survival analyses.

### Statistical analysis

Qualitative variables were compared using *χ*^2^ tests and Fisher’s exact test. For quantitative variables, one-way analysis of variance and Student’s *t*-test were applied for each group. Overall survival was defined as the time from the initial surgery until death from any cause. Survival curves were obtained using the Kaplan–Meier method. Significant differences among subgroups were compared using the log-rank test. The Cox proportional hazard model was used to explore the effects of the clinicopathological variables and other factors. Analyses with *P*-value < 0.05 were considered statistically significant. Statistical analysis was performed using JMP version 11.0.0 (SAS Institute Inc., Cary, NC, USA).

## RESULTS

### Kaplan–Meier survival curves by whole tumour size and solid component size

**Figure 1:**
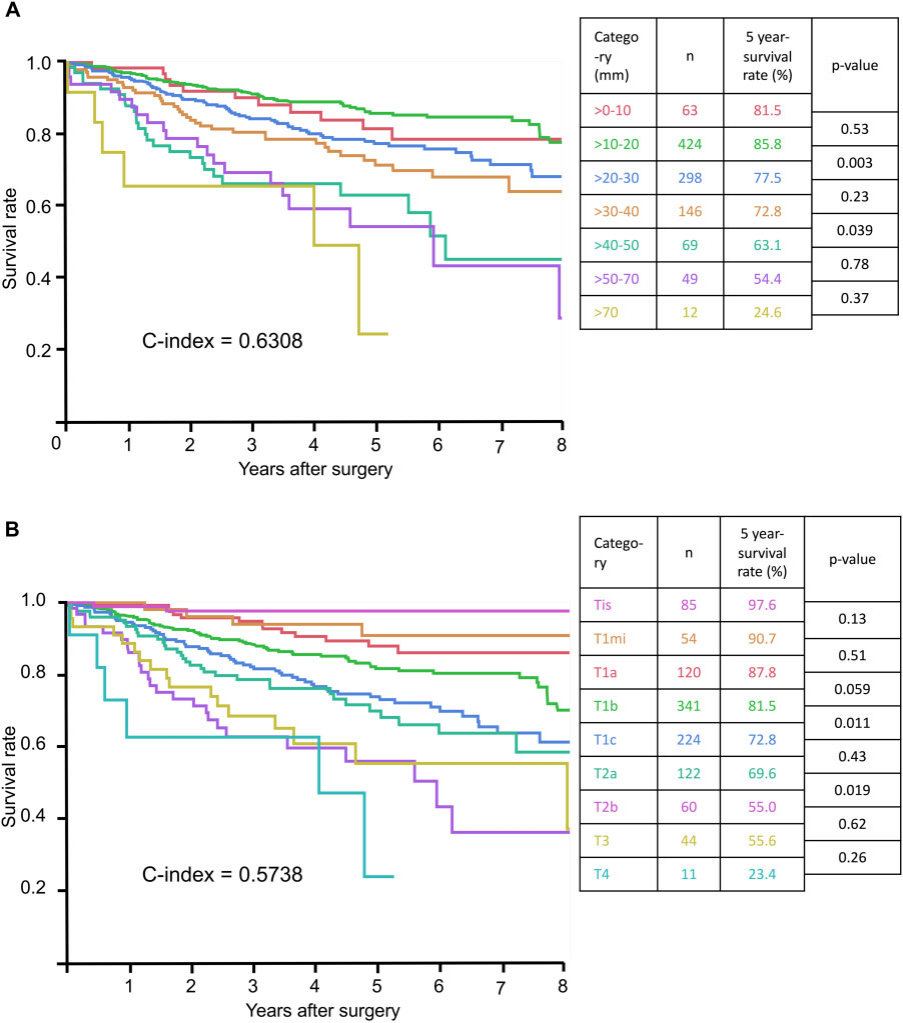
(**A**) Overall survival of all patients according to whole tumour size. (**B**) Overall survival of all patients according to the eighth edition clinical T descriptor.

The Kaplan–Meier survival curves of patients according to whole tumour size and eighth edition cT descriptor are shown in Fig. 1. The postoperative 5-year survival rates (5YS) of patients according to whole tumour size were 81.6% (>0–10 mm, *n* = 63); 85.8% (>10–20 mm, *n* = 424); 77.5% (>20–30 mm, *n* = 298); 72.8% (>30–40 mm, *n* = 146); 63.1% (>40–50 mm, *n* = 69); 54.4% (>50–70 mm, *n* = 49); and 24.6% (>70 mm, *n* = 12). The 5YS of patients according to the eighth edition cT descriptor were 97.6% (cTis, *n* = 85); 90.7% (cT1mi, *n* = 54); 87.8% (cT1a, *n* = 120); 81.5% (cT1b, *n* = 341); 72.8% (cT1c, *n* = 224); 69.6% (cT2a, *n* = 122); 55.0% (cT2b, *n* = 60); 55.6% (cT3, *n* = 44); and 23.4% (cT4, *n* = 11). Although the Kaplan–Meier curves of the new cT descriptor seem to divide the patient prognosis more finely than the whole tumour size in the early T stages, the c-index for the new cT descriptor did not show a better trend than that for the whole tumour size (0.5738 vs 0.6308).

### Demographic and clinicopathological characteristics of patients with ground-glass tumour, part-solid tumour and solid tumour

**Figure 2:**
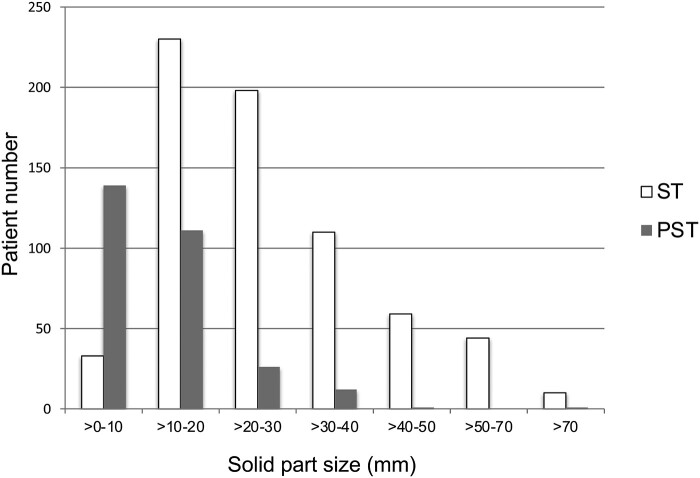
The distribution of the size of the solid part component in patients with ST and PST. PST: part-solid tumour; ST: solid tumour.

The clinicopathological characteristics of all patients are described according to tumour type in Table 1. There were significant differences in sex, surgical procedure, histological findings, whole tumour size, SC size and pathological lymph node metastasis among the 3 groups (*P* < 0.001). Sublobar resection was preferred as the surgical procedure in GGT (67.8%) compared to PST (34.1%) and ST (20.5%). All GGTs and 99% of PSTs were adenocarcinomas which were significantly different from the proportion of STs, 34% of which were non-adenocarcinomas. All cases of GGT and 96% of PST cases had N0 disease, which was significantly higher than the proportion of ST cases (83%) with N0 disease. The distribution of the size of the solid part component in patients with ST and PST is shown in Fig. 2. Ninety-five percent of PSTs had SC diameters of ≤30 mm, whereas 67% of STs had an SC diameter of ≤30 mm; significantly fewer PSTs than STs had an SC diameter >30 mm (*P* < 0.001).

**Table 1:** Clinicopathological characteristics of patients according to tumour type

		Ground-glass tumour (*n* = 87)	Part-solid tumour (*n* = 290)	Solid tumour (*n* = 684)	
Factors	Category	*n* (%)	*n*	*n*	*P*-value
Age (years)	Mean	66.9	68.0	69.2	0.028^a^
		(SD: 8.57)	(SD: 9.24)	(SD: 9.43)	
Sex	Female	32 (36.8)	124 (42.8)	465 (68.0)	<0.001
	Male	55 (63.2)	166 (57.2)	219 (32.0)	
Surgical procedure	Sublobar resection	59 (67.8)	99 (34.1)	140 (20.5)	<0.001
	Lobectomy	28 (32.2)	190 (65.5)	528 (77.2)	
	Pneumonectomy	0 (0)	1 (0.34)	16 (2.3)	
Histological type	Adenocarcinoma	87 (100)	287 (99.0)	435 (63.6)	<0.001
	Squamous	0 (0)	2 (0.7)	195 (28.5)	
	Others	0 (0)	1 (0.3)	54 (7.9)	
Whole tumour size (mm)	>0–10	19 (21.8)	11 (3.8)	33 (4.8)	<0.001
	>10–20	54 (62.1)	140 (48.3)	230 (33.6)	
	>20–30	12 (13.8)	88 (30.3)	198 (29.0)	
	>30–40	2 (2.3)	34 (11.7)	110 (16.1)	
	>40–50	0 (0)	10 (3.5)	59 (8.6)	
	>50–70	0 (0)	5 (1.7)	44 (6.4)	
	>70	0 (0)	2 (0.7)	10 (1.5)	
Solid part size (mm)	0	87 (100)	0 (0)	0 (0)	<0.001
	>0–5	0 (0)	54 (18.6)	1 (0.2)	
	>5–10	0 (0)	85 (29.3)	32 (4.7)	
	>10–20	0 (0)	111 (38.3)	230 (33.6)	
	>20–30	0 (0)	26 (9.0)	198 (29.0)	
	>30–40	0 (0)	12 (4.1)	110 (16.1)	
	>40–50	0 (0)	1 (0.3)	59 (8.6)	
	>50–70	0 (0)	0 (0)	44 (6.4)	
	>70	0 (0)	1 (0.3)	10 (1.5)	
Pathological lymph node metastasis	N0	87 (100)	280 (96.6)	564 (82.5)	<0.001
	N1	0 (0)	4 (1.4)	61 (8.9)	
	N2	0 (0)	6 (2.0)	58 (8.5)	
	N3	0 (0)	0 (0)	1 (0.2)	

a The difference between the ground-glass tumour group and the solid tumour group was significant.

SD: standard deviation.

### Comparison of postoperative survival of ground-glass tumour, part-solid tumour and solid tumour patients according to solid component size

**Figure 3:**
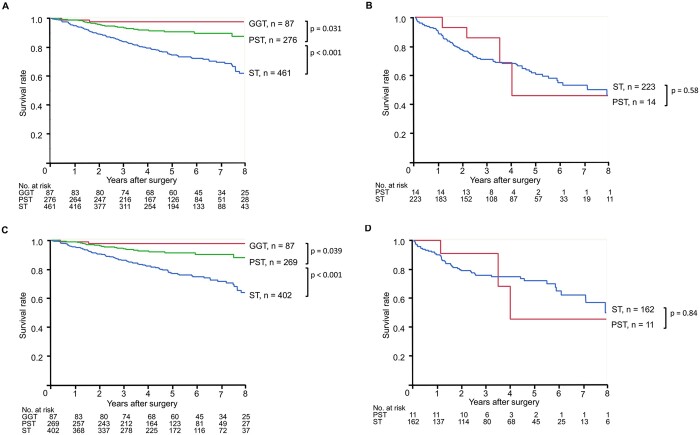
Overall survival of patients stratified according to tumour type: (**A**) patients with a solid component (SC) diameter of ≤30 mm; (**B**) patients with an SC diameter of >30 mm; (**C**) patients with pN0 disease with an SC diameter of ≤30 mm; and (**D**) patients with pN0 disease with an SC diameter of >30 mm. GGT: ground-glass tumour; PST: part-solid tumour; ST: solid tumour.

The postoperative 5YS of NSCLC patients with GGT, PST and ST were 97.6%, 89.0% and 76.3%, respectively. Since the demographics of patients with SC diameter ≤30 and >30 mm were significantly different, we compared the patient survival rates separately among tumours with SC diameters ≤30 and >30 mm. The survival rates between patients with PST and ST among tumours with diameter ≤30 mm were significantly different [Fig. 3A: 5YS, 90.7% vs 74.6%, *P* < 0.001; hazard ratio (HR) 0.33, 95% confidence interval (CI) 0.21–0.51], whereas the difference was not significant among tumours with diameter >30 mm (Fig. 3B: 5YS, 45.7% and 60.4%, respectively, *P* = 0.58; HR 0.75, 95% CI 0.28–2.06). Similar results were also shown in patients with N0 disease (Fig. 3C: 5YS, 90.7% vs 74.6%, *P* < 0.001; HR 0.34, 95% CI 0.22–0.55; Fig. 3D: 5YS, 45.5% and 72.1%, respectively, *P* = 0.84; HR 0.88, 95% CI 0.27–2.85).

### Comparison of postoperative survival of patients with part-solid tumour and solid tumour according to each solid component size

**Figure 4:**
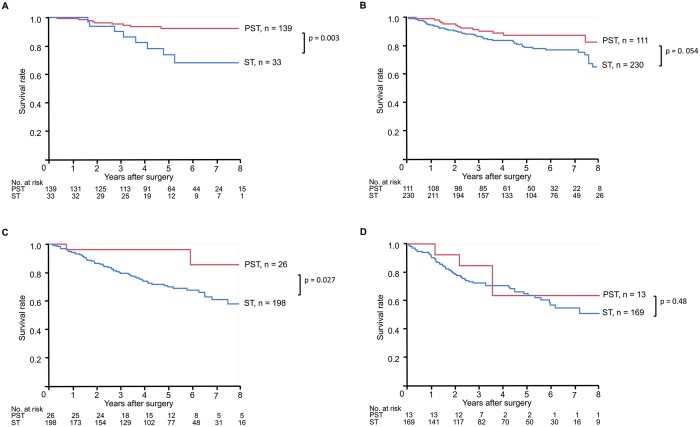
Overall survival of patients stratified according to tumour type: (**A**) patients with a solid component (SC) diameter of >0–10 mm; (**B**) patients with an SC diameter of >10–20 mm; (**C**) patients with an SC diameter of >20–30 mm; (**D**) patients with an SC diameter of >30–50 mm. PST: part-solid tumour; ST: solid tumour.

We further performed survival analyses according to each SC size to eliminate the tumour size imbalance between the PST and ST groups. Postoperative survival of the PST group with tumour diameters ≤10 and 20–30 mm was significantly better than that of the ST group with the same diameters (Fig. 4A: 5YS, 92.2% vs 73.6%, *P* = 0.003; HR 0.26, 95% CI 0.10–0.68; Fig. 4C: 5YS, 96.2% vs 69.8%, *P* = 0.027; HR 0.23, 95% CI 0.06–0.95). The PST group with a tumour diameter of 10–20 mm also tended to have better survival than the ST group with the same diameter (Fig. 4B: 5YS, 87.4% and 78.8%, respectively, *P* = 0.054; HR 0.55, 95% CI 0.30–1.02). No difference in survival was observed among patients with a tumour diameter of 30–50 mm (Fig. 4D: 5YS, 63.7% and 64.8%, respectively, *P* = 0.48; HR 0.66, 95% CI 0.21–2.10).

### Prognostic impact of part-solid tumour on non-small-cell lung cancer

To assess the prognostic impact of PST on NSCLC, we constructed univariable and multivariable Cox regression proportional hazard models. We evaluated the clinicopathological factors among a combination of patients with PST and ST (Table 2, *n* = 974). The multivariable analysis showed that age <70 years, female sex, procedures with a lobectomy or more, SC size, pN0 disease and PST were independent predictors of better survival among all PST and ST patients.

**Table 2:** Results of the univariable and multivariable analyses for predictors of overall survival (the Cox proportional hazard model)

Factors	Category	*n*	Univariable analyses		Multivariable analysis	
			HR (95% CI)	*P*-value	HR (95% CI)	*P*-value
Age (years)	<70	479	Reference	<0.001	Reference	<0.001
	≥70	495	2.20 (1.67–2.92)		2.08 (1.56–2.79)	
Sex	Female	385	Reference	<0.001	Reference	<0.001
	Male	589	2.89 (2.10–3.98)		2.56 (1.84–3.60)	
Surgical procedure	Sublobar resection	239	Reference	0.99	Reference	0.030
	Lobectomy + pneumonectomy	735	1.00 (0.74–1.36)		0.68 (0.49–0.96)	
Histology	Adenocarcinoma	722	Reference	<0.001	Reference	0.80
	Non-adenocarcinoma	252	2.46 (1.87–3.23)		1.04 (0.76–1.42)	
Solid size (mm)	1 mm increase		1.03 (1.02–1.04)	<0.001	1.02 (1.01–1.03)	<0.001
Pathological lymph node metastasis	N0	844	Reference	<0.001	Reference	<0.001
	N1–3	130	2.90 (2.15–3.91)		2.56 (1.85–3.50)	
Tumour type	PST	290	Reference	<0.001	Reference	0.013
	ST	684	3.31 (2.23–4.92)		1.73 (1.14–2.72)	

Statistically significant variables in the univariable analyses as well as clinically significant variables as ‘Surgical procedure’ were used into the multivariable model.

CI: confidence interval; HR: hazard ratio; PST: part-solid tumour; ST: solid tumour.

## DISCUSSION

In the eighth edition of the TNM staging system, the T descriptor was finely divided by 10 mm each for a tumour diameter up to 50 mm, and the tumour size of PST was changed from whole tumour diameter to SC diameter [4, 5]. In addition, pure GGT was defined as Tis (T0), and PST with an SC diameter of ≤5 mm was defined as T1mi. Validation studies have demonstrated that the new T descriptor effectively stratified postoperative patients according to the prognoses [11, 12]. However, Jung *et al.* [13] did not observe the superiority of the eighth edition T descriptor system over that of the seventh edition in their validation analysis, suggesting the need for further studies. The result of the present study supports the superiority of the cT descriptor of the new staging system over that of the seventh edition, with respect to finer stratification of postoperative patients by prognosis.

Since most of the studies that investigated the prognostic impact of PST included only stage IA tumours, data of large-sized PST with SC diameter >30 mm have been limited. In the present study, we demonstrated that only 5% of all PSTs contained tumours with SC diameter >30 mm, in contrast to one-third of STs with SC diameter >30 mm (Fig. 2). These data indicate that the incidence of PST with SC diameter >30 mm was significantly smaller than that of ST with SC diameter >30 mm. We speculate that tumours with a lepidic growth may have less aggressive features that do not grow to a large size in lungs. One report, which included only patients with N0 disease, described a similar proportion; only 3.76% (10 out of 281) of PSTs showed an SC diameter of >30 mm [14]. Nonetheless, to the best of our knowledge, this is the first report to describe the distribution of the SC size of PST among all surgical patients with NSCLC.

Many studies have demonstrated that PSTs with predominant GGC (generally STR ≤ 0.5) are less invasive than PSTs with predominant SC (STR > 0.5) or STs [15]. Recently, even patients with PSTs with predominant SC were shown to have better survival than those with the same size of ST [8, 9]. Hattori *et al.* [14] showed that, regarding PSTs, the new cT descriptor (SC diameter) failed to predict prognosis after surgery, although it worked well with STs. In their study, all PST cases had such good survival regardless of SC size that they could not be stratified according to SC size. In the present study, we demonstrated that among patients with cT1 tumours, those with PST with any SC diameter had better survival than those with ST. This result supports the finding that the new cT descriptor failed to distinguish between patient survival curves among PSTs with small-sized tumours. Conversely, regarding the relatively large tumour with SC diameter >30–50 mm, PST did not show a better prognosis to ST tumours (Fig. 4D), although the number of PST patients in this group was very small (*n* = 13).

Prognostic data of large PSTs with SC size >30 mm have been limited. Recently, Suzuki *et al.* [16] described the clinical features of PSTs with whole tumour diameter exceeding 3.0 cm. This study indicated that PSTs with predominant SC (0.5 < STR < 1) had a worse prognosis than PSTs with predominantly GGC (0 ≤ STR ≤ 0.5); the 5-year overall survival rates were 88.4% and 98%, respectively. Although that study did not show how many PSTs had an SC diameter >30 mm, the average SC diameter of the PSTs with predominant SC was 34 mm. Therefore, many PSTs with predominant SC should have an SC diameter >30 mm. Taken together with the present study, PST patients with SC diameter >30 mm had a worse prognosis than those with small-sized PSTs. We would speculate that as the SC enlarges it may acquire more gene mutations and lose its indolent nature, which may reflect the poorer survival of T2 tumours.

### Limitations 

This study has several limitations. First, it was a retrospective analysis making the study prone to several forms of bias. Nonetheless, to minimize the possible influences from various factors in the survival analysis, we performed a multivariable analysis and demonstrated the prognostic significance of PST with regard to overall survival. In the survival analysis, disease-free survival was not available; which is also important as well as overall survival for patients with early-stage lung cancer who may die from causes unrelated to their lung cancer. Second, a large portion of the surgical patients had incomplete data and were not included in the analysis. The number of patients included in the sub-categorical analyses may have been too small to yield any definitive results. Third, we used several different detectors and different conditions between the 2 hospitals. We did not perform cross validation of the measurements between the 2 hospitals. The use of different measuring procedures of tumour diameter may have influenced the results. We did not have secondary evaluations of CT images by thoracic radiologists. Fourth, detailed pathological information was not available such as subcategories of adenocarcinoma or invasiveness of the tumour, which could not enable us to see the correlation between the clinical and the pathological T descriptor in the study. Fifth, the new staging system was created from an international cohort of patients while this study contained patients from only 2 centres. Regional differences in oncological outcomes may account for some of the shortcomings of the staging system when applied to this patient cohort, especially when comparing small STs and small SCs of PSTs. Accordingly, a well-designed prospective study with a large number of patients is warranted to clarify the prognostic significance of PST in NSCLC.

## CONCLUSIONS

In conclusion, in the detailed analysis of PST and ST for the new cT descriptor, we showed that the incidence of T2a disease or higher is significantly rarer in PST than in ST. PST showed significantly better survival than ST among cT1 tumours, suggesting that the presence of a GGC should be considered in a future improvement of the cT descriptor.

## SUPPLEMENTARY MATERIAL

Supplementary material is available at *ICVTS* online.

### Funding 

This study was supported by a Grant-in-Aid from the Japan Society for the Promotion of Science (# 18K08791 to T.O.).

###  

**Conflict** **of interest:** none declared.

### Author contributions

**Tatsuro Okamoto:** Formal analysis; Funding acquisition; Investigation; Writing—original draft. **Michiyo Miyawaki:** Investigation; Methodology. **Gouji Toyokawa:** Investigation; Methodology. **Takashi Karashima:** Investigation; Validation. **Miyuki Abe:** Resources; Validation. **Yohei Takumi:** Data curation; Validation. **Takafumi Hashimoto:** Investigation; Software. **Atsuhi Osoegawa:** Project administration; Validation. **Tetsuzo Tagawa:** Project administration; Resources. **Hideya Takeuchi:** Resources. **Mototsugu Shimokawa:** Data curation; Formal analysis; Validation. **Kenji Sugio:** Project administration; Writing—review & editing.

### Reviewer information

Interactive CardioVascular and Thoracic Surgery thanks Mohamed Rahouma, and the other anonymous reviewer(s) for their contribution to the peer review process of this article.

## Supplementary Material

ivab255_Supplementary_Data

## ABBREVIATIONS

5YS: 5-Year survival
CI: Confidence interval
cT: Clinical T
CT: Computed tomography
GGC: Ground-glass component
GGT: Ground-glass tumour
HR: Hazard ratio
NSCLC: Non-small-cell lung cancer
PST: Part-solid tumour
SC: Solid component
ST: Solid tumour
STR: Solid-component size-to-whole tumour size
TNM: Tumour, node and metastasis
