# Supplementary material for: Clinical significance of part-solid lung cancer in the eighth edition TNM staging system
Source: Interact Cardiovasc Thorac Surg. 2021 Sep 25;34(2):219–26. doi: 10.1093/icvts/ivab255 (PMC8766204; doi:10.1093/icvts/ivab255)
Supplement: ivab255_Supplementary_Data [file ivab255_supplementary_data.docx]

**Supplementary Table 1.** The patient demographics and clinicopathological characteristics.

| Factors | Category | n | % |
| --- | --- | --- | --- |
| Age | median (range) | 70 | (29-90) |
| Sex | Female  Male | 440  621 | 41.5  58.5 |
| Surgical procedure | Partial or Segmentectomy  Lobectomy  Pneumonectomy | 298  745  17 | 28.1  70.2  1.6 |
| Histology | Adeno  Squamous  Large  Adeno-squamous  Others | 809  197  31  18  6 | 76.2  18.6  2.9  1.7  0.6 |
| Whole tumor size  (mm) | >0-10  >10-20  >20-30  >30-40  >40-50  >50-70  >70 | 63  424  298  146  69  49  12 | 5.9  40.0  28.1  13.8  6.5  4.6  1.1 |
| Solid tumor size  (mm) | 0  >0-5  >5-10  >10-20  >20-30  >30-40  >40-50  >50  >70 | 87  55  117  341  224  122  60  44  11 | 8.2  5.2  11.0  32.1  21.1  11.5  5.7  4.7  1.0 |
| Tumor type | Pure ground class tumor (GGT)  Part-solid tumor (PST)  Solid tumor (ST) | 87  290  684 | 8.2  27.3  64.4 |
| pN | N0  N1  N2  N3 | 931  65  64  1 | 87.7  6.1  6.0  0.09 |
